# Effects of family socioeconomic status on the self-expectations of children under grandparenting in China

**DOI:** 10.3389/fpubh.2025.1479965

**Published:** 2025-02-12

**Authors:** QiXuan Hu

**Affiliations:** School of Political Science and Public Administration, Wuhan University, Wuhan, China

**Keywords:** *g*randparenting, family socioeconomic status, parent–child communication, self-expectation, comparison

## Abstract

**Introduction:**

The increasing expansion of grandparenting necessitates further study of the effects of grandparenting on child development. This study investigated the relationship between family socioeconomic status (SES) and children’s self-expectations in households involving grandparenting, using data from the “China Family Panel Studies” (CFPS). The CFPS is a national, large-scale, multidisciplinary social tracking survey conducted by the Institute of Social Science Survey (ISSS) at Peking University.

**Methods:**

The analysis drew on data from 4,946 children aged 6-16 and their families, collected from CFPS2016 to CFPS2018. To determine whether grandparenting was involved, responses from the Children’s parents’ questionnaire were used. Any caregiving arrangement involving grandparents-whether during the day, at night, or both-was classified as grandparenting. Correlational and hierarchical regression analyses were conducted to examine the association between family SES and children’s self-expectations.

**Results:**

The findings revealed a significant negative correlation between family SES and children’s self-expectations, including its various dimensions, in both groups of families. Additionally, family SES was found to negatively predict children’s self-expectations. A potential explanation for this result is that children from low-income families may have a stronger desire to improve their environmental and social circumstances, fostering greater internal motivation and higher self-expectations. In comparison to families without grandparenting, those with grandparenting had significantly lower family SES, children’s self-expectations, parent-child communication, and parental marital status, with more students studying in non-elite schools. Parent-child communication and residential areas for children can positively predict children’s self-expectations in both groups of families.

**Discussion:**

These findings highlight the significance of family SES and the influence of multiple factors for raising the self-expectations of children under grandparenting. Thus, to improve the quality of life for children under grandparent care and promote their physical and mental health requires a multi-level approach involving the state, society, and individuals within the family.

## Introduction

The family serves as children’s first classroom and is crucial to their growth. Combining elements such as increased life expectancy for grandparents, employment prospects for mothers, the proportion of single-parent households, demographic pressure, and changes in social status highlight the significance of grandparents in childcare. Globally, the percentage of families with grandparents is rising. 9.72% of children live with their grandparents, and 8.20% of children live in families where three generations coexist, per America’s Families and Living Arrangements 2022 (1). According to data from the Survey of Health, Ageing and Retirement in Europe (SHARE), 44% of grandmothers and 42% of grandfathers in Europe frequently or sporadically took care of their grandkids (2).

### The role of grandparenting in family education

Although grandparents are typically not seen as the major caregivers in the family (3), they can participate in raising their grandkids by “grandparenting” (4). Grandparenting, which has been influenced by Chinese traditions, is particularly prevalent in China due to the idea of “intergenerational exchange,” according to which grandparents expect support from their children and grandchildren to ensure an improved standard of living in later years as recompense for their current labor-intensive support of the family. The difficulties in China’s social growth and advancement in recent years have come from the rapid pace of population aging, the declining birth rate, less open positions on the job market, and mounting strain on pensions and the education of only children. The “separate two-child” policy, in place since 2013, the “comprehensive two-child” policy, in place since 2015, and policies relating to the birth of three children, in place since 2021, are just a few of the policies the Chinese government has implemented to deal with the effects of the “aging population” and decline in birth rate. The financial strain on families has increased significantly even though these measures have actively supported the stability of population growth. With the demographic transition, economic evolution, and social transformation in China, the size of families has gradually decreased, and the generational structure has become flattened. These changes have significantly weakened the family’s reproductive and caregiving functions, increasing grandparents’ involvement in child-rearing to compensate for these deficiencies. Between 1990 and 2020, the proportion of standard nuclear families (comprising parents and unmarried children) dropped significantly, from 51.4 to 26.5% (5). This shift highlights the transformation of traditional family structures, with nuclear families no longer dominating. In this context, grandparents have assumed a more prominent role within three-generational family structures, helping to maintain family stability and functionality. Under China’s current employment conditions, parents are required to dedicate substantial time and energy to their work. With overtime becoming commonplace, parents often lack sufficient time to care for their children. For example, young schoolchildren are often dismissed from school earlier in the day, while parents remain at work, making timely pick-ups and drop-offs difficult. Grandparents’ involvement becomes essential in such scenarios, offering not only better care for children but also critical support that enables parents to focus on their work responsibilities.

The “family systems theory” views the family as a network of several subsystems that are connected by reciprocal constraints (6). The contact between family members during the family life cycle creates a dynamic state of intimacy and alienation. In addition to the parent–child relationship, this approach also emphasizes the value of other family interactions (6). Grandparents can therefore help to lessen family and social stress by offering a variety of forms of support so that people or families can successfully handle the strains of modern living. There is evidence that raising grandchildren can, to a certain extent, lessen the employment pressure on mothers, allowing more of them to leave the house and obtain employment (7). From 1991 to 2004, 45% of grandparents in China lived with their grandchildren until they started primary school and spent the same amount of time each week caring for them as their mothers do (8). According to a survey done by the China Aging Science Research Center, 66.5% of Chinese families now include grandparents (4). Grandparenting may have an impact on children’s emotions, behaviors, cognitive development, and academic success, according to previous studies on the mental health, life satisfaction, and cognitive function of grandparents (9–13). Therefore, grandparenting has an impact on children’s development in addition to being associated with family socioeconomic status (SES).

### Family SES and children’s self-expectations

Academic achievement and mental health are two areas of a child’s development that are widely acknowledged to be significantly predicted by family SES (14, 15). In literature, it has been defined and assessed in a variety of ways (16, 17), with three main components of education, income, and occupation being the most frequently taken into account. In the current study, family SES is considered a complete measure of family annual income, family education level, and family occupational level.

Children from homes with higher SES typically have superior academic attainment, access to more resources and opportunities, according to previous research (18, 19). As Kraus et al. (83) noted, the thinking patterns and perceptions of the world of people with high-SES are characterized by freedom, choice, internal drive, control, and a prioritization of individual considerations, it follows that different family SES levels have an impact on children’s future perspectives and behaviors. Additionally, the Wisconsin model of status attainment emphasizes that individual self-expectations can have an impact on educational achievement in addition to external objective factors (20).

Family background, culture, and social resources all have an impact on an individual’s self-expectations, which include the hope of obtaining a higher social status (20). The urge to learn is stronger when one’s expectations are higher (21). Expectations are broken down into outcome expectations and self-efficacy expectations by Bandura (22). The individual’s judgments about the results of their acts, as well as the hopes and beliefs they hold about the results, make up their outcome expectations. People will act responsibly because they believe certain outcomes will result from their activities. Expectation serves as a powerful motivator for students and helps them to control their learning processes and actions (23). Therefore, the following Hypothesis was proposed:

H1: The self-expectations of children under grandparenting are affected by family SES. The higher the family SES, the more likely the children will set higher self-expectations.

### The influence of multiple factors on children’s self-expectations

#### Grandparenting

According to Sewell et al. (20), many variables can affect children’s self-expectations, including cultural norms, family support, and family structure. According to Sear and Coall (24), grandparenting is one of the most important sources of support for families. Additionally, it has been claimed that seasoned grandparents can support their grandchildren’s cognitive development by creating a better learning environment for them or by teaching them through their own words and actions (25). Moreover, children who live with their grandparents perform better academically and have superior communication skills (10, 26), raising the self-expectations of the grandchildren.

However, other studies showed that grandparenting not only produces anxiety and insecurity in children but also causes developmental delay and split personalities (27, 28). Grandparenting has also been found to lower the quality of life for grandparents and cause parents to spend less time with their kids as they grow up (29, 30). These studies showed that grandparenting has blatantly detrimental consequences on children’s development and lowers children’s expectations for their future development. The academic achievement of children may be impacted differently by various types of grandparenting (31). The academic achievement of grandchildren may be negatively impacted by grandparents with low educational backgrounds (32). Therefore, there is an apparent lack of consensus on the role of grandparenting in children’s self-expectations, and further research into the important influencing elements is necessary. The following hypothesis was proposed:

H2: Family SES and children’s self-expectations in families involved grandparenting are significantly lower than those without grandparenting.

Less research has been done on the impact of grandparents on children’s self-expectations, particularly among those from lower SES families, despite studies showing that grandparents have a positive impact on grandchildren’s education in low-income families (33, 34). Grandparenting has a broad impact on two dimensions of expectations for grandchildren’s achievement. One is the legacy effect, or direct influence of grandparents on their grandchildren, which includes not only the explicit provision of material or emotional resources to grandchildren but also the implicit influence from grandparents, i.e., obtaining family networking resources by residing with grandparents (34). The second aspect is the indirect impact of grandparents on their grandchildren, which is passed on to their parents through the grandparents’ education. For instance, the impact of grandparents’ SES and educational orientation on parents’ educational expectations, which in turn increases the parents’ educational expectations on grandchildren’s educational expectations (35), consolidating grandparents’ long-term “idioculture” of achievement (36). As a result, the educational outcomes of kids who had grandparents as role models significantly correlate with both their parents and their grandparents.

#### Parent–child communication

The current study aims to further explore the potential factors by which parents affect the self-expectation of children who receive grandparenting. It cannot be ignored that one of the most crucial elements for children’s social development and self-awareness is communication between parents and children (37). In particular, the sharing of knowledge, thoughts, feelings, or attitudes between parents and children can help to resolve issues and fortify emotional ties (38). According to research, parents with middle-class or higher SES are more likely to verbally interact with their kids, show more emotion, use more authoritative parenting styles, and engage in “collaborative training” with their kids (39). However, several recent studies have also shown that class barriers in China are primarily caused by external factors like money and that there are no class differences when it comes to parents’ attitudes toward raising their children, the development of children’s non-cognitive abilities, or self-negotiation education (40–42).

It has also been discovered that communication between parents and children affects kids’ expectations of themselves. The social cognitive theory states that interactions between parents and their children might affect their self-assessment of how they can do a given task (22). Parents typically communicate their beliefs, interests, and expectations to their children and send more positive messages to their children, which will boost their confidence and inspire higher self-expectations (43). The family social capital theory also highlights the value of parents’ involvement, supervision, and care for their kids (44). Coleman proposed that parents can properly foster their children’s general growth only with the help of good emotions like love and trust. As a result, a key component influencing children’s self-expectations is parent–child communication, one of the representations of parental education participation. Children from families with high SES are more likely to receive encouragement and support from parents and other significant others, as well as having quick access to more resources and information, benefiting from parents’ focus on and emphasis on education, thus strengthening children’s self-expectations (45). According to Sewell and Shah (46) and Sewell and Shah (47), factors including family SES, parental encouragement of their children, and students’ IQ levels will considerably and favorably influence their hopes for attending elite colleges.

A few prior studies have explored the impact of parent–child communication on children under grandparenting, even though there is a positive correlation between parent–child communication and children’s mental and physical health (37, 48). As a result of the limited time available for direct communication and exchange between parents and their children in grandparenting families, children raised by grandparents often have estranged relationships with their parents and face many adaptation challenges, including loneliness, vulnerability, anxiety, and depression as they grow up and experience more serious psychological problems than children raised by their parents (49). To effectively raise children’s self-expectations and better support their development in grandparenting families, it is vital to investigate if parent–child communication can predict children’s self-expectations, especially these under grandparenting. Therefore, the following hypotheses were proposed:

H3: Parent-child communication in families involving grandparenting is significantly lower than that in families without grandparenting.

H4: Parent-child communication can positively predict children’s self-expectations in both groups of families.

#### Children’s living environment

Living conditions not only affect physical health, but also have a significant impact on mental well-being (50). Compared with adults, children are more susceptible to the influence of the external environment. Most of the places where children live and study are fixed whether the place of residence is in an urban or rural area, or whether the school is elite. These living environments will become one of the important factors affecting children’s mental health. Studies have shown that there is a large gap in educational resources obtained by Chinese children living in cities and rural areas (51). Factors such as uneven distribution of basic education resources and key schools have solidified urban–rural education inequality. In addition, due to the continuous and cumulative nature of education, access to higher education opportunities faces layers of selection. There is obvious “path dependence” in this differentiated selection process, i.e., the quality of education a student receives at a certain stage depends on the type of school he attended in the previous stage (52), among them, the most obvious diversion effect is between elite schools and non-elite schools. Research shows that students who have attended elite schools have a significantly higher final average educational level than students from non-elite schools (53). Some studies have evaluated the long-term impact of the elite school system in the basic education stage on individual income and further pointed out that after 1993, Chinese students’ attendance in elite junior high schools had a significant impact on their future income (54). Attendance in elite junior high schools also affects an individual’s subsequent educational achievements and thus their income in adulthood. It can be seen that where a child lives and the types of schools he attends will inevitably have an impact on his future development and, in turn, his own level of self-expectations. Therefore, the following hypotheses were proposed:

H5: Attendance in elite schools in families without grandparenting is higher than families involving grandparenting.

H6: Good residential area positively predicts children’s self-expectations in both groups of families.

In addition to the physical living environment, the family atmosphere shaped by the relationship between parents also affects children’s growth and development. Good marital quality of parents is an important factor in maintaining family stability. It not only affects the status of the couple themselves, but also affects the growth of their children (55). Bronfenbrenner’s (56) ecological systems theory emphasizes that the different experiences of family members jointly shape the ecological environment and atmosphere of the family, which in turn will affect the developmental trajectories of each family member (57). The interdependence and influence in Family systems theory suggests that there is an important link between the parents’ sense of competence and marital functioning in the parenting subsystem (6), and marital quality in the marriage subsystem helps to promote the development of the parent–child subsystem (58). Parents with high marital quality tend to express more positive emotions in the family (59). Open communication and emotional expression make it easier for parents to understand their children’s needs, provide guidance and encouragement to their children, perform better parenting roles, and enhance the intimate relationship with their children (59, 60). Thus, parents’ satisfaction with marriage will have an impact on their parenting ability to a certain extent (61), which will affect the development of children’s self-expectations. Therefore, the following hypotheses were proposed:

H7: Parental marital status in families without grandparenting is better than families involved grandparenting.

#### The present study

Although Bradley and Corwyn (14) and Chevalère et al. (15) reported that family SES was substantially associated with children’s academic performance, mental health, and educational aspirations, little research has been done on the relationship between family SES and the self-expectations of children raised by grandparents, including academic and behavioral self-expectations. Therefore, based on the Wisconsin model of status attainment (20) and family systems theory (6), the study assumed that grandparenting, parent–child communication and children’s living environment could affect the influence of family SES on the self-expectations of children.

The Wisconsin model of status attainment, developed by American sociologist William Hamilton Sewell and colleagues, builds upon and refines the original status attainment model. This model emphasizes the critical role of family background, particularly SES, in shaping an individual’s educational expectations and outcomes. In this study, family SES was analyzed through dimensions such as annual family income, educational attainment within the family, and occupational levels. The Wisconsin model expands on the Blau-Duncan model by incorporating psychosocial factors, including intelligence, academic achievement, influence from significant others, career aspirations, and educational aspirations. These additions refine the understanding of how family background affects educational and vocational outcomes, offering a more comprehensive explanatory framework. Within this context, academic performance emerges as a crucial dimension of the theory. Several studies have demonstrated a strong correlation between executive function and academic achievement (62, 63). Moreover, individual motivation is influenced by the expectation of success and the perceived value of tasks, with individuals who hold high self-expectations more likely to exert greater effort, thereby increasing their likelihood of success. Based on these theoretical foundations, this study divides children’s self-expectations into two categories: academic self-expectations and behavioral self-expectations. Additionally, the Wisconsin model underscores the importance of a family’s resource accessibility. Factors such as the area where children reside and the schools they attend serve as proxies for a family’s resource access, making these variables key components in the analysis.

The family systems theory highlights the impact of emotional interdependence and interaction patterns among family members on individual behavior and psychological states. This theory organizes families into subsystems, including the spousal subsystem, parent–child subsystem, and sibling subsystem, each with distinct roles and tasks. The boundaries and interaction patterns between subsystems significantly influence family functioning. When these boundaries become blurred, family dynamics may suffer. Consequently, dimensions such as parent–child communication and parental marital status were incorporated into the study.

Additionally, the multigenerational transmission process, as outlined in this theory, provides valuable insights into the impact of grandparenting on children’s development. This process emphasizes how behavioral patterns, emotional responses, and relationship dynamics are transmitted across generations. Understanding these patterns enables individuals to break unhealthy cycles and foster healthier relationships for themselves and future generations. This study, therefore, focused on examining the influence of family SES on children’s self-expectations in households with and without grandparenting involvement. The study had two primary objectives: (1) To examine the concurrent associations between family SES, children’s self-expectations (both academic and behavioral), parent–child communication, residential area score, attendance at elite schools, and parental marital status in two family groups. (2) To test the impact of various factors on children’s self-expectations through hierarchical regression analysis, particularly analyzing the role of family SES while controlling for other variables.

## Data source and basics

This study has used the database from the China Family Panel Studies (CFPS) conducted by the Institute of Social Science Survey of Peking University (ISSS). The database comprises multi-phase follow-up surveys on many families in 25 provinces (autonomous regions and municipalities) in mainland China except Qinghai, Ningxia, Inner Mongolia, Tibet, Hainan, and Xinjiang. The surveys cover the family income and expenditure, family living conditions, education of each adult family member, family health condition and job situation, and the daily life, education, and training of each child in the family. The research has been carried out regularly all year round, and all data collections have been carried out with the approval of the Biomedical Ethics Committee of Peking University. 4 types of the CFPS questionnaire have been used: community questionnaire, family questionnaire, adult self-answered questionnaire (interviewees aged 16 and above), and questionnaire for Children’s Parents (Adolescents aged 0–15). The database used in this study consists of three parts: CFPS2016 and CFPS2018 family questionnaires, adult self-answered questionnaires, and a questionnaire for Children’s Parents. Socio-demographic information was gathered from all questionnaires, while family questionnaires were used to obtain data on family SES. Additionally, adult self-answered questionnaires and questionnaires completed by children’s parents provided data on children’s self-expectations and related influencing factors.

**Figure 1:**
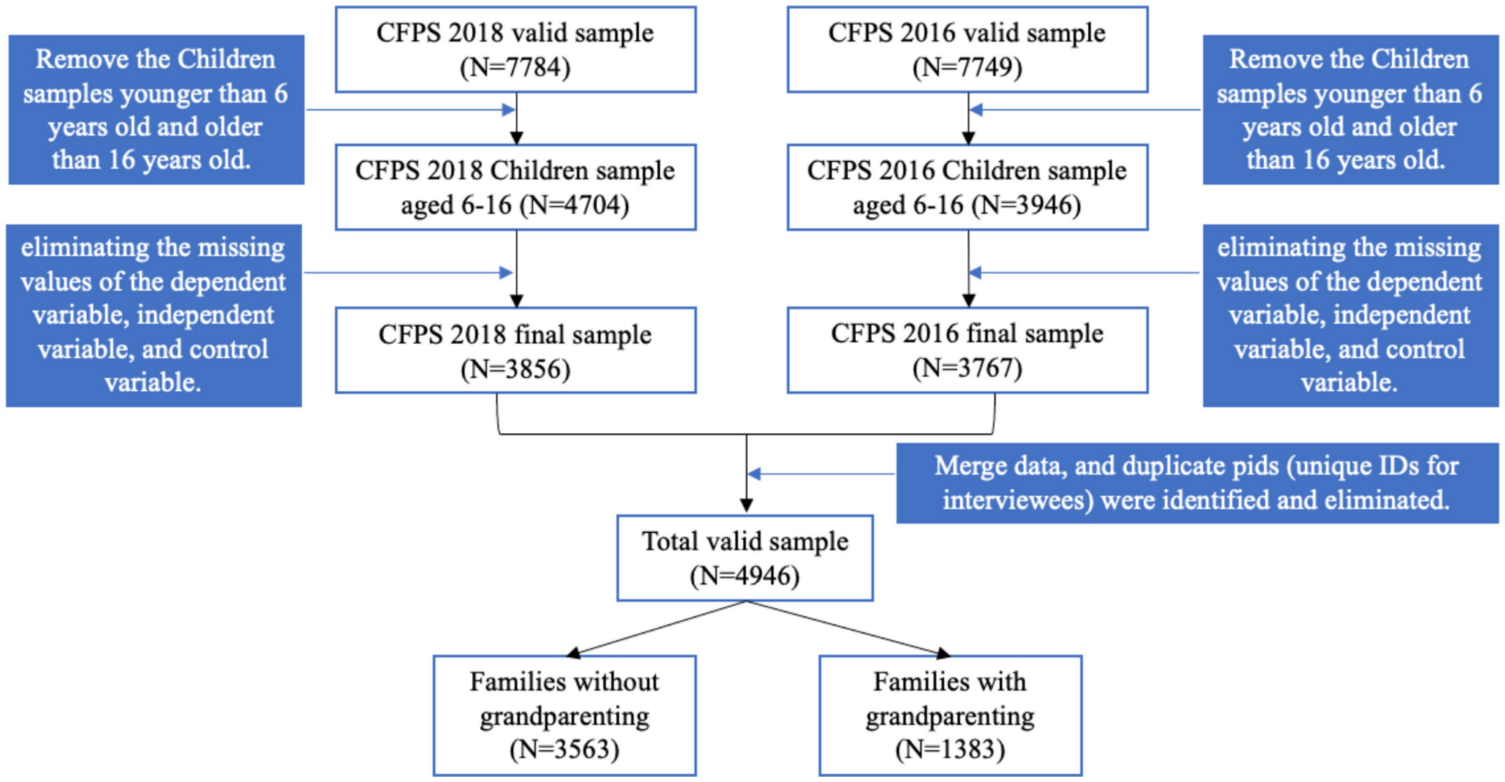
The selection process of research sample.

The CFPS 2018 and CFPS 2016 datasets were processed in the following sequence and criteria (as outlined in Figure 1):

1. To focus on school-age children, samples of children younger than 6 years old or older than 16 years old were excluded from the total valid sample.
2. Cross-sectional data were created by matching the datasets of parents and their children (aged 6–16) using parent–child relationships identified in the family relationship database.
3. Cases with missing values for dependent, independent, or control variables were removed.
4. Data from CFPS 2018 and CFPS 2016 were merged, and duplicate pids (unique IDs for interviewees) were identified and eliminated.

As a result, 4,946 valid datasets were selected for analysis. These datasets were divided into two groups based on the presence or absence of grandparenting: (1) Families without grandparenting: 3,563 cases (72.04%). (2) Families with grandparenting: 1,383 cases (28.96%). Among the 4,946 samples with a mean age of 10.48 years, there were 2,315 females (46.81%) and 2,631 males (53.19%). 4,089 children (82.67%) were from rural regions and 849 children (17.17%) were from urban areas, and the information on present districts for 8 children (0.16%) was missing. 268 parents (5.40%) were not in marriage or any forms of serious relationships, 4,678 parents (94.60) were in a stable relationship. 3,724 students (75.29%) were not in elite schools, 1,222 students (24.71%) were in elite schools.

## Variables of measurement

### Family SES

The family SES uses five measurement indicators: family economic situation, father’s education level, mother’s education level, father’s occupation level, and mother’s occupation level. These five indicators are further combined to generate three parameters: family annual income, family education level, and family occupation level. The family annual income is derived from the logarithm of the family annual income. According to the questionnaire options, the family education level is reclassified into 8 educational levels from 1 to 8, which are (1) no formal education, (2) elementary school, (3) junior high school, (4) high school/technical secondary school/Technical school/vocational high school, (5) junior college, (6) undergraduate, (7) master, (8) doctorate degrees. The parent with a higher educational level is used as the index calculation basis, and the score represents the educational level of the family. If there is only one parent providing information, it will be used as the basis for the family’s education level score.

Based on the CFPS occupational coding rules^1^, I divided parents’ occupations into 6 categories, i.e., (1) working in agriculture, forestry, animal husbandry, fishery, and water conservancy, (2) production and transportation equipment operators and related personnel, (3) working in the business and service sectors, (4) Clerks and related personnel, (5) professional skill workers, (6) in charge of state organizations, party-mass organizations, enterprises, and institutions. Scores from 1 to 6 were given based on the occupational level, and the higher the score, the higher the occupational level. The parents with higher occupational levels were taken as the basis for index calculation, and the score represents the family’s occupational social status.

I then converted the three indicators of the two types of samples into standard scores and conducted principal component analysis based on related research (64). In the sample of families practicing grandparenting, a main factor with a characteristic root greater than 1 was obtained, explaining 49.54% of the variance while the main factor with the obtained characteristic root greater than 1 explained 48.74% of the variance in the sample of families without grandparenting. Since both groups obtained characteristic roots were all greater than 1, only the coefficient of the main factor needed to be presented. The calculation formula of the SES index of the family with grandparenting is defined as:


$$\begin{array}{l} \mathrm{family}\;SES_{\mathrm{with}\;\mathrm{grandparenting}}=(0.703\times Z_{\mathrm{family education level}}+\\ 0.724\times Z_{\mathrm{family occupation level}}+0.684\times Z_{\mathrm{family annual income}})/1.486 \end{array}$$

The calculation formula of SES indicators for families without grandparenting is defined as:


$$\begin{array}{l} \mathrm{family}\;SES_{\mathrm{without}\;\mathrm{grandparenting}}=(0.613\times Z_{\mathrm{family education level}}\\ +0.727\times Z_{\mathrm{family occupation level}}+0.747\times Z_{\mathrm{family annual income}})/1.462 \end{array}$$

The numbers 0.703, 0.724, 0.684 in the SES index represented the factor loads of three indexes, respectively, in the families associated with grandparenting while 1.486 was the characteristic root of the principal factor; while in families without grandparenting, the numbers were 0.613, 0.727, 0.747 and the characteristic root of the principal factor was 1.462. The range of SES indexes for families associated with grandparenting is 3.67–12.63 and the range of SES indexes for families without grandparenting is 2.67–13.40. The higher the value, the higher the family SES score.

### The self-expectations of children

The current study focused on the self-expectations of children in primary and junior high schools. According to the Wisconsin model of status attainment (20), the 7 scale items related to children’s self-expectations in the children’s parents’ questionnaire were used, which were further divided into two dimensions: academic self-expectation and behavioral self-expectation, and each dimension was divided into a 5-point scoring system: strongly disagree, disagree, neutral, and agree, strongly agree. Since mothers generally spend more time with their children, the index calculation score was mainly based on the mother’s score. The academic self-expectation dimension contains 3 items: (1) the child studies very hard; (2) the child will double-check homework after completion; and (3) the child will only play after completing the homework. In the samples of families with and without grandparenting, the Cronbach’s *α* were 0.790 and 0.772, respectively. The behavioral self-expectation dimension contains 4 items: (1) the child is focused during studies; (2) the child obeys school rules; (3) once the child starts something, it must be finished; and (4) the child is very organized. In the samples of families with and without grandparenting, the Cronbach’s α were 0.780 and 0.788, respectively.

### Grandparenting

Based on three questions in the children’s parents’ questionnaire: “Who will take care of the child during the day,” “Who will take care of the child at night” and “Who usually picks up and drops off the child,” it is determined whether there is grandparenting involved. If one of the three items chose to be taken care of or picked up by “grandfather/grandmother,” it was determined to be a sample of families engaging in grandparenting; those who chose to be taken care of or picked up by other people for all three items were considered as samples of non-grandparenting.

### Parent–child communication

Parent–child communication was assessed by using the questionnaire for Children’s Parents. The item “Parents actively communicate with their children” is a neutral item based on the interviewer’s observation of the interviewee’s family relationship. By adopting a 5-point scale, parent–child communication was divided into 5 levels: strongly disagree, disagree, neutral, agree, and strongly agree. The index calculation score was mainly based on the mother’s score.

### Choice of other variables

(1) Score of child’s residential area; the score is based on the question “What is the child’s current household registration status?” in the questionnaire for Children’s Parents, with 0 representing “an agricultural household registration” and 1 representing a “non-agricultural household registration.” (2) Attendance status in elite schools. Whether the child attends an elite school comes from the question “Is the child attending an elite school?” in the questionnaire for Children’s Parents, with 0 representing “no” and 1 representing “yes.” (3) Parental marital status. The determination of parents’ marital status comes from the question “What is your current marital status?” in the adult self-answered questionnaire. 0 means “not in marriage or any serious relationships,” and 1 means were “in a stable relationship”.

## Missing data handling

In this study, case deletion and missing value imputation methods were used to handle missing data. Among the variables determined by the relevant groups, the deletion method was adopted for any missing items, such as data lacking basic information such as children’s gender and age, and data missing key grouping variables. The one-variance test method was used to confirm that the above variables were completely missing at random. For the cases with only one item missing, the missing value was replaced by the mean interpolation method, and there was no significant difference in other key variables before and after imputation. By using these processing methods, the sample size of children from families with grandparenting was 1,383, and the sample size of children from families without grandparenting was 3,563.

## Statistical analyses

Descriptive statistics was employed to examine the means and standard deviations for gender, age, parent–child communication, SES (family annual income, family education level, family occupation level), self-expectation (academic self-expectation and behavioral self-expectation), score of child’s residential area, attendance status in elite schools, and parental marital status. Tables 1, 2 present the means, standard deviations, independent sample *t*-test result and correlations for all the observed variables. Independent sample *t*-test was applied to examine the differences between families with and without grandparenting for all research variables. The Correlational analysis was used to preliminarily exam the degree of association between variables. Variables significantly related to the self-expectations of children were included in hierarchical regressions to examine the influence of predictors on the self-expectations of children with and without participation in grandparenting. Finally, variables were gradually incorporated into the hierarchical regression model to detect the regression analysis results of family SES after controlling other variables.

**Table 1:** Descriptive statistics and Independent-sample *t*-test among all the observed variables.

Variables	No grandparenting M	SD	Grandparenting M	SD	*t*
Gender of child	0.530	0.499	0.530	0.499	0.043
Age of child	11.01	2.933	9.120	2.636	21.968^***^
Family SES	8.370	1.512	8.276	1.442	2.027^*^
Parent–child communication	2.819	1.056	2.691	0.965	4.067^***^
Score of child’s residential area	0.170	0.374	0.180	0.386	−1.127
Attendance status in elite schools	0.255	0.436	0.223	0.416	2.007^*^
Parental marital status	0.960	0.199	0.910	0.283	5.571^***^
Academic self-expectations	8.736	2.862	8.261	2.858	5.234^***^
Behavioral self-expectations	11.651	3.655	11.014	3.576	5.535^***^
The total score of self-expectations	20.387	6.087	19.275	6.025	5.780^***^

*N* = 4,946. ^*^ *p* < 0.05, ^**^ *p* < 0.01, ^***^p < 0.001.

**Table 2:** Interrelations among all the observed variables of families with and without grandparenting.

Variables	1	2	3	4	5	6	7	8	9	10
Gender of child		0.014	0.059^*^	0.066^*^	−0.019	0.018	0.024	0.043	0.049	0.050
Age of child	−0.005		0.056^*^	0.075^**^	−0.068^*^	0.557^**^	−0.073^**^	0.009	−0.018	−0.006
Parent–child communication	0.005	0.138^**^		−0.010	−0.117^*^	0.031	−0.208^**^	0.321^**^	0.373^**^	0.374^**^
Score of child’s residential area	0.003	0.019	−0.011		−0.014	0.074^**^	0.395^**^	0.032	0.096^**^	0.072^**^
Parental marital status	0.001	−0.036^*^	−0.023	−0.028		−0.045	0.115^**^	−0.015	−0.017	−0.018
Attendance status in elite schools	−0.052^**^	0.334^**^	0.001	0.053^**^	−0.042^*^		−0.073^**^	0.022	0.017	0.020
Family SES	0.018	−0.106^**^	−0.176^**^	0.375^**^	0.091^**^	−0.010		−0.103^**^	−0.094^**^	−0.105^**^
Academic self-expectations	0.032	0.078^**^	0.360^**^	0.068^**^	−0.043^**^	0.000	−0.111^**^		0.751^**^	0.920^**^
Behavioral self-expectations	0.024	0.099^**^	0.442^**^	0.055^**^	−0.033	0.035^*^	−0.128^**^	0.741^**^		0.950^**^
The total score of self-expectations	0.029	0.096^**^	0.435^**^	0.065^**^	−0.040^*^	0.021	−0.129^**^	0.915^**^	0.949^**^	

The data above the table are for families involved grandparenting and the data below the table are for families without grandparenting. *N* = 4,946. ^*^ *p* < 0.05, ^**^ *p* < 0.01.

## Results

### Preliminary analyses

The results of descriptive statistics and Independent-sample *t*-test were presented in Table 1.

Independent-sample *t*-test result showed that the total scores of the age of the child, family SES, parent–child communication, parental marital status, attendance status in elite schools and children’s self-expectation of families without grandparenting were significantly higher than those of families involving grandparenting. There is no significant difference in the scores of gender and child’s residential area between families with and without grandparenting.

In addition, there was no significant gender difference between the two groups of families. Families with or without grandparenting showed no significant gender differences in the total score of self-expectations (including academic self-expectations and behavioral self-expectations) and family SES. However, there were significant gender differences in parent–child communication (M_females_ = 2.63, SD_females_ = 0.93, M_males_ = 2.74, SD_males_ = 0.99, *t* = −2.21, *p* < 0.05) and score of child’s residential area (M_females_ = 0.15, SD_females_ = 0.362, M_males_ = 0.21, SD_males_ = 0.404, *t* = −2.48, *p* < 0.05) in families with grandparenting. Specifically, the parent–child communication for males (M = 2.74) was significantly higher than that for females (M = 2.63), and the score of child’s residential area for males (M = 0.21) was also significantly higher than that for females (M = 0.15). Whereas there were no significant gender differences reported in parent–child communication (M_females_ = 2.81, SD_females_ = 1.08, M_males_ = 2.82, SD_males_ = 1.04, *t* = −0.27, *p* > 0.05) or score of child’s residential area (M_females_ = 0.17, SD_females_ = 0.373, M_males_ = 0.17, SD_males_ = 0.375, *t* = −0.16, *p* > 0.05) of the families without grandparenting.

The results of interrelations among all the observed variables of samples in different groups were shown in Table 2.

The Correlational analysis results of the two groups of families displayed the same characteristics as follows: There was a significant negative correlation between family SES and the total score of self-expectation and its dimensions. The age of child was significantly negatively correlated with family SES. Parent–child communication and family SES were both significantly and negatively correlated, whereas parent–child communication had a significant positive correlation with the age of child and the total score of self-expectation and its dimensions (both academic self-expectations and behavioral self-expectations). Besides, there was a significant positive correlation in all dimensions of self-expectations. Moreover, the score of child’s residential area was significantly and positively correlated with attendance status in elite schools and family SES. Parental marital status was significantly and negatively correlated with the ages of children but was significantly and positively correlated with family SES. The attendance status in elite schools was significantly and positively correlated with the ages of the children. The age of child was also significantly positively correlated with parent–child communication in families with grandparenting, but it was significantly negatively correlated with family SES.

In addition, in families involved grandparenting, the gender of child was significantly positively correlated with parent–child communication and the score of child’s residential area. The score of child’s residential area was significantly and positively correlated with children’s behavioral self-expectations and the total score of self-expectations. In addition, parental marital status was significantly and negatively correlated with parent–child communication, and the attendance status in elite schools was also significantly and negatively correlated with family SES.

Although In families without grandparenting, the gender of child was significantly and negatively correlated with the attendance status in elite schools, the age of child was significantly positively correlated with the total score of self-expectation and its dimensions. Parental marital status was significantly and negatively correlated with the attendance status in elite schools, children’s academic self-expectations, and the total score of self-expectations. Attendance status in elite schools was also significantly and positively correlated with children’s behavioral self-expectations, but was significantly and negatively correlated with child’s gender. Moreover, among families without grandparenting, the score of child’s residential area was also significantly and positively correlated with children’s academic self-expectations.

### The hierarchical regression analysis

From the above correlation analysis, it was found that the correlation between the gender of child and attendance status to elite schools and the total score of children’s self-expectations was not significant in both groups of families, so these variables were not entered to the hierarchical regression model. Hierarchical regression analysis can effectively verify the independent contributions of the age of child, score of child’s residential area, parental marital status, parent–child communication and family SES to children’s self-expectations. The variables that were significantly correlated with the total score of self-expectation of the two groups of families in the correlation analysis were included in the hierarchical regression model as independent variables, and hierarchical regression analysis was performed in groups. The order of variables entering the model started with demographic and sociological information and ends with family SES to present the regression analysis results of family SES after controlling other variables. The independent variables were arranged in the following order to verify their independent contribution: (1) The age of child; (2) Score of child’s residential area; (3) Parental marital status; (4) Parent–child communication; and (5) Family SES.

Table 3 shows hierarchical regression results for families involved grandparenting. The variance inflation factor (VIF) values ranged from 1.000 to 1.272 (VIF < 10), which indicates that there is no multicollinearity. The result of The Durbin-Watson test (1.710) has verified the independence between observed values in this study. First, when the age of child was included in the regression model, the result showed no significance (*F* = 0.05, *p* > 0.05). Secondly, when the score of child’s residential area was entered, the regression model was significant (*F* = 3.71, *p* < 0.05). The results showed that when grandparenting was involved, the score of child’s residential area significantly and positively predicted children’s self-expectations (*β* = 1.14, *p* < 0.01). Thirdly, when parental marital status was included into the regression model. The model was significant (*F* = 2.61, *p* < 0.05), but the prediction result of parental marital status on children’s self-expectations was not significant (*β* = −0.37, *p* > 0.05). Fourthly, after controlling the above background variables, it was found that parent–child communication was a significant predictor variable that significantly and positively predicted children’s self-expectations (*β* = 2.28, *p* < 0.001). The regression model was significant (*F* = 59.50, *p* < 0.001), with the predictors explaining 15% of the variance in children’s self-expectations. Finally, after adding the predictor variable of family SES, it was found that family SES significantly and negatively predicted children’s self-expectations (*β* = −0.33, *p* < 0.01). The final regression model included the above five predictor variables, and the model was significant (*F* = 49.45, *p* < 0.001; *R^2^* = 0.15, *p* < 0.01).

**Table 3:** Hierarchical regression results for families involved grandparenting.

Predictors	Step 1		Step 2		Step 3		Step 4		Step 5	
	*B*	*β*	*B*	*β*	*B*	*β*	*B*	*β*	*B*	*β*
Constant	19.40^***^		19.31^***^		19.67^***^		12.83^***^		15.73^***^	
Age of child	−0.01	−0.01	−0.03	−0.01	−0.03	−0.01	−0.07	−0.03	−0.09	−0.04
Score of child’s residential area			1.14^**^	0.07^**^	1.14^**^	0.07^**^	1.23^**^	0.79^**^	1.73^**^	0.11^**^
Parental marital status					−0.37	−0.02	0.55	0.03	0.70	0.03
Parent–child communication							2.37^***^	0.38^***^	2.28^***^	0.37^***^
Family SES									−0.33^**^	−0.08^**^
*R^2^*	0.00		0.01^**^		0.01		0.15^***^		0.15^**^	
*ΔR^2^*	0.00		0.01		0.00		0.14		0.01	
*F*	0.05		3.71^*^		2.61^*^		59.50^***^		49.45^***^	
*ΔF*	0.05		7.37		0.42		228.87		8.01	

*N* = 1,383. ^*^ *p* < 0.05, ^**^ *p* < 0.01, ^***^p < 0.001.

Table 4 shows hierarchical regression results for families without grandparenting. The VIF values ranged from 1.000 to 1.231 (VIF < 10), which also indicates that there is no multicollinearity. The result of the Durbin-Watson test (1.486) has verified the independence between the observed values. Firstly, when the age of child was included in the regression model, the results show that the regression model was significant (*F* = 33.05, *p* < 0.001). In families without grandparenting, the age of child had a significant and positive predictive effect on children’s self-expectations (*β* = 0.02, *p* < 0.001). Secondly, when the score of child’s residential area was included in the regression model. The model was still significant (*F* = 23.79, *p* < 0.001), and the predictors explained 13% of the variance in children’s self-expectations. The score of child’s residential area had a significant and positive predictive effect on children’s self-expectations in families without grandparenting (*β* = 1.03, *p* < 0.001). Thirdly, when parental marital status was included in the regression model. The model was significant (*F* = 17.34, *p* < 0.001). Parental marital status had a significant and negative predictive effect on children’s self-expectations (*β* = −1.07, *p* < 0.05). Fourthly, when parent–child communication was incorporated into the regression model, it significantly and positively predicted children’s self-expectations (*β* = 2.48, *p* < 0.001). The regression model was significant (*F* = 216.31, *p* < 0.001), with the predictors explaining 20% of the variance in children’s self-expectations. Finally, after controlling for other variables, family SES was included in the hierarchical regression model, and it was found that family SES could significantly and negatively predict children’s self-expectations (*β* = −0.36, *p* < 0.01). The final regression model included the above five predictor variables, and the model was significant (*F* = 180.72, *p* < 0.001; *R^2^* = 0.20, *p* < 0.001).

**Table 4:** Hierarchical regression results for families without grandparenting.

Predictors	Step 1		Step 2		Step 3		Step 4		Step 5	
	*B*	*β*	*B*	*β*	*B*	*β*	*B*	*β*	*B*	*β*
Constant	18.20^***^		18.05^***^		19.11^***^		13.21^***^		16.30^***^	
Age of child	0.20^***^	0.10^***^	0.20^***^	0.10^***^	0.20^***^	0.10^***^	0.07^*^	0.03^*^	0.06	0.03
Score of child’s residential area			1.03^***^	0.06^**^	1.01^***^	0.06^***^	1.11^***^	0.07^***^	1.67^***^	0.10^***^
Parental marital status					−1.07^*^	−0.04^*^	−0.82	−0.03	−0.57	−0.02
Parent–child communication							2.48^***^	0.43^***^	2.40^***^	0.42^***^
Family SES									−0.36^**^	−0.09^**^
*R^2^*	0.01^***^		0.13^***^		0.01^*^		0.20^***^		0.20^***^	
*ΔR^2^*	0.01		0.00		0.00		0.20		0.01	
*F*	33.05^***^		23.79^***^		17.34^***^		216.31^***^		180.72^***^	
*ΔF*	33.05		14.41		4.40		803.88		29.12	

*N* = 3,563. ^*^ *p* < 0.05, ^**^ *p* < 0.01, ^***^p < 0.001.

## Discussion

Children’s self-expectations play a crucial role in their overall development. Although the current study focuses on family SES, grandparenting and relevant factors, many other internal and external factors can shape children’s expectations and influence how they perceive their abilities, set goals, and form expectations for themselves. For instance, parental influence (46, 47), social environment (65), peer relationships (66) and past experiences (67). Whether and how these key factors affect the impact of family SES and grandparenting on children’s self-expectations requires further investigation. Although it is well-recognized that grandparenting impacts the development of children (10, 31, 49), potential factors affecting the relationship between family SES and the self-expectations of children under grandparenting are poorly understood. To fill this gap, the current study focusing on families with grandparenting, revealed how family SES impacted children’s self-expectations and further investigated potential factors which influence the expectations of children under grandparenting. The study highlighted the significance of family support in forming children’s self-expectations by comparing families with and without grandparenting to shed light on the potential effects of grandparenting in the lives of children from various socioeconomic backgrounds.

Hierarchical regression analysis revealed that, after controlling for other variables, family SES significantly and negatively predicted children’s self-expectations in both groups of families. This finding contrasts sharply with prior research suggesting a significant positive correlation between family SES and children’s academic achievement and career aspirations (15, 19), thereby refuting Hypothesis 1. Heyneman and Loxley (68) claimed that there is no significant relationship between a student’s achievement and their family background in less developed nations because of the lack of access to education opportunities that will increase their drive to learn. Indeed, 82.67% of the samples included in the current study came from remote villages in China. Another potential explanation for this result is that children from low-income families may have a stronger desire to improve their environmental and social circumstances, fostering greater internal motivation and higher self-expectations. Previous research has demonstrated that students’ perceptions of socioeconomic class have a considerable impact on their academic performance (19). Strong self-expectations can motivate kids from low-income homes to achieve more (69). Children’s self-expectations can fill up the gaps left by their family’s SES. As a result, Milne and Plourde (70) found that children’s internal motivation, the impact of how they perceive their family’s SES, and their strong desire to change their future position (i.e., to have higher self-expectations) are all important factors in the good academic achievement attained by children from low SES homes.

The Independent-sample *t*-test results revealed that the age of child, family SES, parent–child communication, parental marital status, attendance status in elite schools, and children’s self-expectations were all significantly lower in families involved grandparenting than in families without grandparents. This finding lends support to Hypothesis 2, which may be connected to the fact that grandparents play a significant compensatory role in their grandchildren’s development, particularly in families experiencing adversity such as divorce, the death of a parent, or financial hardship (33). Grandparenting is more prevalent among low-income families in China than high-income families (71), which may be related to the fact that families with high SES can afford to hire nannies or confinement maids to take care of children to prevent conflicts with their grandparents regarding educational concepts and lifestyles. The current study found that grandparenting lowers children’s self-expectations in both academic and behavioral aspects, which is consistent with earlier studies showing that kids in families with grandparents typically perform worse academically and have more behavioral issues (28, 72).

According to Deindl and Tieben (32) and Nanthamongkolchai et al. (11), grandparents’ educational attainment has a significant impact on how well their grandchildren develop in both academics and behavioral habits in families where grandparents play a role in raising the children. Additionally, the independent *t*-test also suggests that compared to families without grandparenting, the level of parent–child communication is significantly lower in families with grandparenting, which is consistent with the fact that parents frequently leave their children with grandparents in many rural areas of China when they are working hard to make ends meet in the city (73). Thus, hypothesis 3 was supported. Children who live closer to their grandparents are more likely to talk to them about their feelings because grandparents spend more time caring for their grandkids (74). It is important to note that communication between parents and children in homes with grandparenting is much higher for boys than for girls, so as the score of child’s residential area. This phenomenon may be related to the cultural distinctions of the Chinese “patriarchal” concept. Grandparenting may increase the likelihood of this happening in China, where the patriarchal idea is more serious and there is a traditional concept of continuing incense and handing along the family line (75). Grandparents who subscribe to the conventional idea of “raising children for future support from children when they get old” will invest more of their family’s resources in their grandsons, which in turn will have an impact on the parents’ attitudes toward their grandsons and cause parents to focus more on their sons.

Prior research has demonstrated that, within the same family SES, children who communicate with their parents more frequently perform better academically (43). The present study also suggests that parent–child communication can positively predict children’s self-expectations in families both with and without grandparenting, which supports hypothesis 4. The result indicates that parent–child communication, which Jackson et al. (76) identified as a core and fundamental factor influencing children’s psychological development, has a favorable effect on raising children’s self-expectations. Even if higher family SES can cause a reduction in children’s self-expectations, better parent–child communication can nevertheless significantly raise children’s self-expectations. Refers to previous research, parents without access to educational resources may increase emotional investment by communicating more with their kids and monitoring and promoting their learning, in order to compensate the lack of material investment (77). Parental emotional commitment may be more successful in preventing the harm brought on by poverty (78). Thus, parent–child communication in families involved grandparenting can significantly contribute to raise children’s self-expectations, indicating that parent–child communication may play a crucial role in preserving the growth of children’s self-expectations.

The results show that compared with children in families with grandparenting, children in families without grandparenting are more likely to attend elite schools, which supports hypothesis 5. Based on the prevalence of grandparenting in low-income families (33), children in families involving grandparenting may have more difficulties in accessing high-quality educational resources. Prior studies have reported that students from high SES families are more likely to enter elite schools in the early education stages and obtain better educational resources and better educational opportunities, which forms an “accumulation of advantages” and ultimately leads to inequality in educational attainment (79). There are differences between elite schools and non-elite schools in terms of teaching staff, infrastructure, financial support, etc. Elite schools also have priority in support from local governments. Therefore, more educational resources are allocated to elite schools, and students in elite schools will enjoy better learning conditions. Published studies have found that elite schools will attract better teachers, and knowledgeable and insightful teachers can better support students and provide students with timely guidance, and help students to improve self-expectations (80), however, not everyone has the opportunity to enter elite schools, especially students living in the rural areas. For instance, in primary schools and junior high schools in China, the admission system of elite schools is closely related to the residential areas. Based on the implementation of the “school district system,” a child’s household registration location determines the primary school and junior high school the child will attend. The results of hierarchical regression show that the score of child’s residential area significantly and positively predicts children’s self-expectations in both groups of family samples, thus supporting hypothesis 6. In this study, the score of child’s residential areas are based on agricultural household (rural area) registration and non-agricultural household (urban area) registration. The urban–rural dual system was formed in the early days of the founding of New China. This system invisibly gave birth to a dual economic pattern in which urban and rural areas were “separated from each other.” In recent years, a large number of rural residents have flowed into cities and towns, causing schools in rural areas to be demolished or merged due to insufficient student resources. As a result, the imbalance in the distribution of urban and rural educational resources has been exacerbated. However, the upper social class can influence the results of educational diversion through direct intervention in the choice of schools (53) to ensure that their children can obtain better educational resources. For example, they can choose schools for their children through “household registration transfer” or purchasing real estate in the residential areas of elite school districts where their children have more access to high-quality educational resources, which has a positive impact on their construction of self-expectations.

Consistent with a previous study (81), the independent-sample *t*-test result showed that parental marital status in families without grandparenting is better than families involved grandparenting. Therefore, hypothesis 7 is supported. Published studies have shown that partner support and marriage maintenance behaviors in the family are considered important predictors of parenting competence (82). Parents with high marital quality know how to support and understand each other, maintain good marital behaviors, and are more likely to accept and recognize their goals and expectations as parents. By guiding their own awareness of the role of parents and fulfilling corresponding responsibilities can also play a better guiding role in the future growth of their children. Compared to children with grandparenting, children who live in families without grandparenting have more opportunities to get along with their parents directly and are more positively affected by the close relationship with their parents.

Given the above conclusions, improving the quality of life for children under grandparent care and promoting their physical and mental health requires a multi-level approach involving the state, society, and individuals within the family.

From the national level, governments and enterprises should implement family-friendly policies, such as paid parental leave and flexible working arrangements, to reduce the burden on grandparents and increase parent–child interaction time. For example, in October 2024, the General Office of the State Council of the People’s Republic of China issued the Act on *Several Measures to Accelerate the Improvement of the Childbirth Support Policy System and Promote the Construction of a Childbirth-Friendly Society*. This policy outlines measures to support childbirth services, build childcare systems, and enhance education, housing, and employment conditions. Key initiatives include extending marriage, maternity, nursing, and parental leave, raising maternity allowance standards, and simplifying the application process for such benefits. These financial support policies aim to reduce families’ economic burdens and improve children’s quality of life.

From the societal level, community centers and non-profit organizations can play a critical role in transforming traditional grandparenting practices. Offering training courses on topics such as child psychological development, nutrition and health, and safety education can equip grandparents with modern parenting concepts and skills. Additionally, making online resources available ensures easier access to such knowledge. These efforts can enhance grandparents’ understanding of effective childcare practices and align their approaches with contemporary needs.

From the family level, within the family, fostering good communication and providing emotional support are essential for children’s social development and self-expectations. Parents should prioritize spending quality time with their children to strengthen communication and connection. Similarly, grandparents should maintain regular communication with parents to share insights into children’s daily needs, educational goals, and physical and mental well-being. Emotional support from parents and grandparents can boost a child’s sense of security and enhance self-expectations. Furthermore, grandparents, as custodians of family history and cultural heritage, can contribute to children’s sense of belonging and identity. Sharing stories about the family’s background and the country’s history can help instill positive values and shape a strong sense of identity in children.

## Limitations and implications

Several limitations of the present study should be noted. The item design is not entirely focused on the relationship between family SES and multiple factors which could influence children’s self-expectations, and the sample size from rural areas is also excessive, even though this study uses rich, mixed, national, and comprehensive CFPS dataset to accurately reflect the reality of Chinese society. Therefore, follow-up studies should create survey items that are more in accordance with the interaction of the three variables and conduct comprehensive surveys in a balanced manner in urban and rural locations. Additionally, the study was conducted only in the context of Chinese culture, whether the conclusion can be generalized to other cultural backgrounds is not clear. Therefore, additional research is required to examine the problem in a multicultural setting and reveal the influence of underlying cultural or group differences through cross-cultural comparative research.

With the increase in grandparenting families, the mental health of children in families engaging in grandparenting needs urgent attention. This study uses hierarchical regression analysis in order to more comprehensively and thoroughly analyze the impact of different influencing factors on children’s self-expectations. By controlling other factors, we can draw a clear conclusion that family SES can indeed affect the self-expectations of children in both groups of families. Family SES and children’s self-expectations of families with grandparenting are significantly lower than those of families without grandparenting, showing the complexity of families with grandparents involved in education and its negative impact on children’s own development. Children receiving grandparenting may experience greater difficulties because of the uncertain factors brought on by grandparenting. These difficulties include loneliness, vulnerability, anxiety, and depression, all of which are detrimental to the development of children’s physical and mental health (49). The current study also highlights the potential value of parent–child communication in affecting the self-expectations of children under grandparenting. As parent–child communication can positively predict children’s self-expectations, parents in grandparenting families should spend more time communicating with their children, thereby promoting their children’s physical and mental well-being to have a favorable effect on the growth of their self-expectations. Moreover, the residential area where children live will also have a positive prediction effect on the development of their self-expectations. Based on this, the country should further introduce corresponding policy that regulates and ensures the balance of access to educational resources for children living in the city and rural area. In addition, the study also found that there are significant differences between the two groups of family samples in terms of attendance status in elite schools and parental marital status. Families without grandparenting perform better in these two dimensions. Moreover, in the hierarchical regression model of families with grandparenting, model 3 is significant when parental marital status is included, but after incorporating more variables, the dimension of parental marital status is no longer significant. In response to this phenomenon, we will further explore the impact of marital status on children’s self-expectations and its causal relationship with grandparenting in future studies.

## Data Availability

The original contributions presented in the study are included in the article/supplementary material, further inquiries can be directed to the corresponding author.

## Footnotes

^1^The occupational codes of the CFPS 2018 baseline survey were based on the “National Standard Occupational Classification and Codes of the People’s Republic of China” (GB/T6565-2009) and were further revised by referring to the occupational and industry classification standards of the “China Social Tracking Survey.” For details, see https://www.isss.pku.edu.cn/cfps/docs/20180927133129327096.pdf
